# P-663. Evaluating the Effectiveness of a Real-Time Electronic Alert to Monitor the Duration of Antibiotic Therapy in Hospitalized Pneumonia Patients in the Asian Healthcare Setting

**DOI:** 10.1093/ofid/ofae631.860

**Published:** 2025-01-29

**Authors:** Sanju Biju, Shon Paulson

**Affiliations:** St. Joseph's College of Pharmacy, Thrissur, Kerala, India; St. Joseph's College of Pharmacy, Thrissur, Kerala, India

## Abstract

**Background:**

Limiting the duration of antibiotic prescribing to the shortest effective period is recognized as a strategy to mitigate the risk of antibiotic resistance. This study aims to assess the impact of a Real-Time Electronic Alert system designed to monitor the duration of antibiotic therapy in hospitalized pneumonia patients, with the goal of reducing unnecessary antibiotic exposure.

**Methods:**

The intervention involved the implementation of a dashboard capable of generating real-time alerts whenever hospitalized patients diagnosed with community- or hospital-acquired pneumonia received antibiotics for more than five consecutive days. The study was conducted between December 2022 and January 2024 at two medical college hospitals in South Asia. Alerts generated by the dashboard were regularly reviewed by an antibiotic stewardship (AS) team, who intervened when patients exceeded the guideline-recommended duration of antibiotic therapy for pneumonia without additional clinical indications warranting continued antibiotic use. To evaluate the effectiveness of the intervention, the inappropriate duration of antibiotic therapy was compared before and after the implementation of the dashboard. A similar hospital with comparable patient demographics served as a comparative group.

**Results:**

During the intervention period, the AS team reviewed a total of 218 patients flagged by the dashboard, resulting in 117 documented interventions. Reasons for lack of intervention included the presence of additional infection diagnoses necessitating prolonged antibiotic therapy, co-infections, and delayed clinical improvement. Post-implementation, the mean excess duration of antibiotic therapy for pneumonia decreased to 0.48 days compared to the pre-implementation mean of 2.13 days. In contrast, the comparative hospital, which did not employ the alert system, experienced a post-intervention mean excess duration of 1.97 days compared to a pre-intervention mean of 2.04 days.

**Conclusion:**

The pneumonia dashboard represents a potentially valuable stewardship tool for reducing unnecessary antibiotic exposure in pneumonia patients.

**Disclosures:**

**All Authors**: No reported disclosures

